# Downregulation of ammonium uptake improves the growth and tolerance of *Kluyveromyces marxianus* at high temperature

**DOI:** 10.1002/mbo3.1290

**Published:** 2022-05-25

**Authors:** Yi Ai, Tongyu Luo, Yao Yu, Jungang Zhou, Hong Lu

**Affiliations:** ^1^ State Key Laboratory of Genetic Engineering, School of Life Sciences Fudan University Shanghai P.R. China; ^2^ Shanghai Engineering Research Center of Industrial Microorganisms Fudan University Shanghai P.R. China; ^3^ Shanghai Collaborative Innovation Center for Biomanufacturing (SCICB) East China University of Science and Technology Shanghai P.R. China

**Keywords:** ammonium uptake, adaptive laboratory evolution, *Kluyveromyces marxianus*, thermotolerance, reactive oxygen species

## Abstract

The growth and tolerance of *Kluyveromyces marxianus* at high temperatures decreased significantly in the synthetic medium (SM), which is commonly used in industrial fermentations. After 100 days of adaptive laboratory evolution, a strain named KM234 exhibited excellent tolerance at a high temperature, without loss of its growth ability at a moderate temperature. Transcriptomic analysis revealed that the KM234 strain decreased the expression of the ammonium (NH_4_^+^) transporter gene *MEP3* and increased the synthesis of the amino acid carbon backbone, which may contribute greatly to the high‐temperature growth phenotype. High NH_4_^+^ content in SM significantly increased the reactive oxygen species (ROS) production at high temperatures and thus caused toxicity to yeast cells. Replacing NH_4_^+^ with organic nitrogen sources or increasing the concentration of potassium ions (K^+^) in the medium restored the growth of the wild‐type *K. marxianus* at a high temperature in SM. We also showed that the NH_4_^+^ toxicity mitigated by K^+^ might closely depend on the *KIN1* gene. Our results provide a practical solution to industrial fermentation under high‐temperature conditions.

## Graphical abstract

Uptake of excess ammonium promotes the production of reactive oxygen species and adversely impacts the growth of *Kluyveromyces marxianus*. Downregulation of ammonium uptake improves the growth and tolerance of *K. marxianus* at high temperatures. Ammonium toxicity in *K. marxianus* mitigated by potassium may depend on the *KIN1* gene.

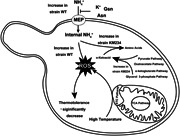

## 1 INTRODUCTION

The growth ability of yeast at high temperatures is a critical phenotype for the industrial fermentation of ethanol, food products, and other useful metabolites. Especially, ethanol fermentation at high temperatures, for example within a 40°C–50°C range, is expected to achieve simultaneous saccharification and fermentation, which can effectively reduce the cooling costs and the risk of contamination (Limtong et al., 2007). However, heat tolerance is a polygenic phenotype, and the current research is far from completely understanding it (Huang et al., 2018). Heat shock proteins (HSPs) and chaperone proteins have been documented to be essential for yeast survival at high temperatures (Lertwattanasakul et al., 2015). In some cases, accumulating trehalose (Matsumoto et al., 2018) and decreasing lipid peroxidation (Mejía‐Barajas et al., 2017) are well‐known practical ways to improve the high‐temperature tolerance of yeast. Although high‐temperature tolerance is a property of industrial preference for yeast, previous studies on it were commonly carried out in yeast extract peptone dextrose medium (YPD), not in synthetic medium (SM) that is mostly used in industrial applications. As far as we know, only Caspeta et al. (2014) carried out the study in SM with an unnatural thermotolerance of *Saccharomyces cerevisiae* as the starting strain.

In YPD medium, peptone and yeast extract are the organic nitrogen sources. Due to the high cost, these nitrogen sources are rarely used in industrial production. As an alternative, ammonium salts, such as ammonium sulfate, ammonium chloride, and ammonium phosphate, are preferable nitrogen sources for large‐scale fermentation production, which are inorganic and cheaper nitrogen sources conducive to reducing the production cost (Marini et al., 2006).

*Kluyveromyces marxianus* is a natural thermotolerant strain that can grow at 45°C or above (Fu et al., 2019; Matsumoto et al., 2018), which makes it a valuable tool for exploring the heat tolerance genes (Htg^+^) and elucidating the heat tolerance mechanisms (Lertwattanasakul et al., 2015). It is also generally recognized as a safe (GRAS) yeast that has proven to be a promising eukaryotic microbe for industrial applications (Leonel et al., 2021), such as the production of single‐cell oil, fatty acids (Karim et al., 2020), lignocellulolytic enzymes (Zhou et al., 2018), and *β*‐mannanase (Pan et al., 2011), as well as efficient preparations of virus‐like particles vaccines (Yang et al., 2021; Duan et al., 2019). However, to our knowledge, the thermotolerance of *K*. *marxianus* in SM has not been well studied yet.

The growth of *K*. *marxianus* decreased significantly at high temperatures in the industrial media using ammonium sulfate as the nitrogen source, which impedes its applications in protein expression and ethanol fermentation. Therefore, to improve its thermotolerance we adaptively evolved *K. marxianus* in SM at temperatures higher than 43°C. After 100 days of adaptive laboratory evolution (ALE), a mutant strain KM234 with significantly improved tolerance at 45°C was obtained. Meanwhile, it also showed no obvious change in growth at 30°C. To study the mechanism underlying the adaptive response to inorganic sources at high temperatures, the transcriptome was sequenced, and the result revealed that, in the KM234 strain, transcription of the genes that were involved in the carbon metabolism, multiple amino acid carbon skeleton synthesis pathways, NH_4_^+^ uptake, and transportation were significantly changed in response to high temperature. In addition, the substitution of NH_4_^+^ with glutamine or asparagine and competition of NH_4_^+^ transport with K^+^ in SM demonstrated that a high concentration of NH_4_^+^ decreased the thermotolerance and growth of *K. marxianus* at high temperatures, which is probably due to the large production of reactive oxygen species (ROS). A high ratio of K^+^ */*NH^+^ can alleviate the NH_4_^+^ toxicity to *K. marxianus* at high temperatures, and this phenotype may be closely associated with the existence of the *KIN1* gene.

## 2 MATERIALS AND METHODS

### 2.1 Yeast strains and experimental evolution

The *K. marxianus* strain FIM‐1 used in this study has been described previously (Wu et al., 2020). Laboratory cultures of *K. marxianus* strains were carried out in flasks shaking at 220 rpm, and cell densities were determined by measuring the optical density (OD) at 600 nm (OD_600_). For ALE, the FIM‐1 strain was cultured in 50 mL SM at 45°C for 24 h with shaking at 220 rpm. Every 24 h, the cultures were transferred into 50 mL fresh SM at an initial OD_600_ of 0.3 and grew under the same conditions as described above. The entire ALE process lasted 100 days. To screen thermotolerance improved clones, the culture of 100 days ALE was serially diluted and spread on YPD plates. After being cultured at 30°C for 24 h, single clones were picked out and their growth phenotypes were tested in flasks containing 50 mL SM shaking at 220 rpm at 30°C and 45°C, respectively.

### 2.2 Culture medium

The standard sythetic medium contained 10 g/L glucose, 5 g/L ammonium sulfate, 3 g/L potassium dihydrogen phosphate, 0.244 g/L anhydrous magnesium sulfate, 1 mL/L 1000× vitamin, 2 mL/L 500× trace elements (Zhou et al., 2018). Preparations of SM containing different concentrations of ammonium or potassium were carried out by adjusting (NH_4_)_2_SO_4_ or KH_2_PO_4_ to disable amounts, while the other components remain unchanged. YPD medium contains 10 g/L yeast extraction, 20 g/L glucose, and 20 g/L poly‐peptone.

### 2.3 Analytical methods

Glucose, ethanol, and glycerol were measured on an Agilent 1260 high‐performance liquid chromatography (HPLC). The analytical method for glucose and glycerol; column: ZORBAX NH2 (250 mm × 4.6 mm, 5 μm; Agilent); column temperature: 30°C; RID temperature: 35°C; flow rate: 1 mL/min; elution time: 30 min; mobile phase: 90% acetonitrile. The analytical method for alcohol: guard column: MetaCrab 87H (50 mm  × 4.6 mm; Agilent); column: MetaCrab 87H (300 mm × 7.8 mm; Agilent); column temperature: 35°C; RID temperature: 35°C; flow rate: 0.6 mL/min; elution time: 30 min; mobile phase: 0.01 N H_2_SO_4_ (0.29 mL concentrated sulfuric acid per liter).

### 2.4 DNA extraction and resequencing

Genomic DNA was extracted using the EZNA Fungal DNA kit (Omega Bio‐Tek). Resequencing was carried out by the Illumina pair‐end sequencing (PE150). The reference genome, as well as the gene annotation information including the functional annotation and gene family, were downloaded from the NCBI genome ID10898. The single nucleotide polymorphisms (SNPs) and short insertions and deletions (InDels) were detected by GATK “HaplotypeCaller” function.

### 2.5 RNA extraction and sequencing

After being grown in SM at 30°C or 45°C for 8 h, yeast cells were collected by centrifugation at 5000 *g* at 4°C for 5 min. Total RNA was extracted by the quick RNA Fungal/Bacterial Miniprep Kit (Zymo Research) according to the kit instruction manual. RNA‐seq was performed on an Illumina HiSeqTM2500 at the Chinese National Human Genome Center in Shanghai. To analyze the differential expressed genes (DEGs), the number of reads from three samples was converted into RPKM (reads per kilobase of transcript per million reads mapped), and then used MARS model (MA‐plot‐based method with the random sampling model) in the DEGseq package to calculate the gene expression difference. The false discovery rate (FDR) value that was <0.001 was considered a significant difference. For Gene Ontology (GO) enrichment analysis, hypergeometric distribution was used to calculate the significant enrichment of genes in each GO category with significant expression differences relative to all genes. Gene ontology with FDR ≤0.001 was defined as significantly enriched in differentially expressed genes.

### 2.6 Gene deletions in *K. marxianus*

The *KIN1* deletion in *K. marxianus* was performed by homologous recombination with the aid of a CRISPR plasmid according to the method described previously (Liu et al., 2018). Briefly, gRNA was inserted into *Sap* I site of pUKD‐N122‐AUC, obtaining the plasmid pUKD‐N122‐AUC/Kin1Del. Upstream and downstream homologous fragments of the *KIN1* gene were amplified using the primer pairs UhfKIN1F/UhfFKIN1R and DhfKIN1F/UhfKIN1R from the genome of *K. marxianus*, respectively*.* The two amplified fragments were fused by PCR using the primers UhfKIN1F and UhfKIN1R, and then the amplified product was co‐transformed with the CRISPR plasmid into *K. marxianus* according to the method by Antunes and de Souza Junior (2000). Transformants were selected on hygromycin plates (20 g/L poly‐peptone, 20 g/L glucose, 10 g/L yeast extraction, 200 μg/mL hygromycin, and 20 g/L agar), and verified with the primers KIN1DvF and KIN1DvR. Primers and gRNA described above were listed in Table 3.

### 2.7 Qualitative and quantitative determination of ROS and GSH

Determination of ROS was performed using 2',7'‐dichlorofluorescein diacetate (H2DCFDA) (D6883; Sigma) as the substrate. Briefly, yeast cells grown in SM for 8 h at 45°C were collected by centrifugation, washed with PBS twice, and suspended in PBS at OD_600_ = 10. After that, 0.5 μL cell suspensions were taken for staining with H2DCFDA according to the kit instruction manual. Wide‐field fluorescence intensity was imaged on an Olympus ix83 inverted microscope equipped with an oil immersion objective (150x/1.45; Olympus) and an EMCCD camera (Evolve 512; Delta Photometrics) using the following filters (Semrock) combinations: excitation filter (BP457–487), dichroic mirror (495LP), and emission filter (BP 502.5–37.5). The gain of EMCCD is 60, and the exposure time is 100 ms.

Glutathione (GSH) was quantified using a BC1175 Micro Reduced GSH Assay Kit (Solarbio).

### 2.8 Statistical and graphical analysis

Statistical analysis was performed in GraphPad‐Prism version 8.0 software (GraphPad Software). Significant differences were examined by the following procedures: (1) Test if the data follow a normal distribution. (2) If the data do not follow the normal distribution, the nonparametric *t*‐test Mann–Whitney test was used. (3) If the data follow the normal distribution, the data satisfying the homogeneity of variance were tested. In the *F*‐test, a *p*‐value > 0.1 is considered to have homogeneity of variance. (4) If the data had homogeneity of variance, the unpaired *t*‐test was used, otherwise, Welch's corrected unpaired *t*‐test was introduced.

## 3 RESULTS

### 3.1 Growth phenotype of *K. marxianus* and ALE at high temperatures

**FIGURE 1:**
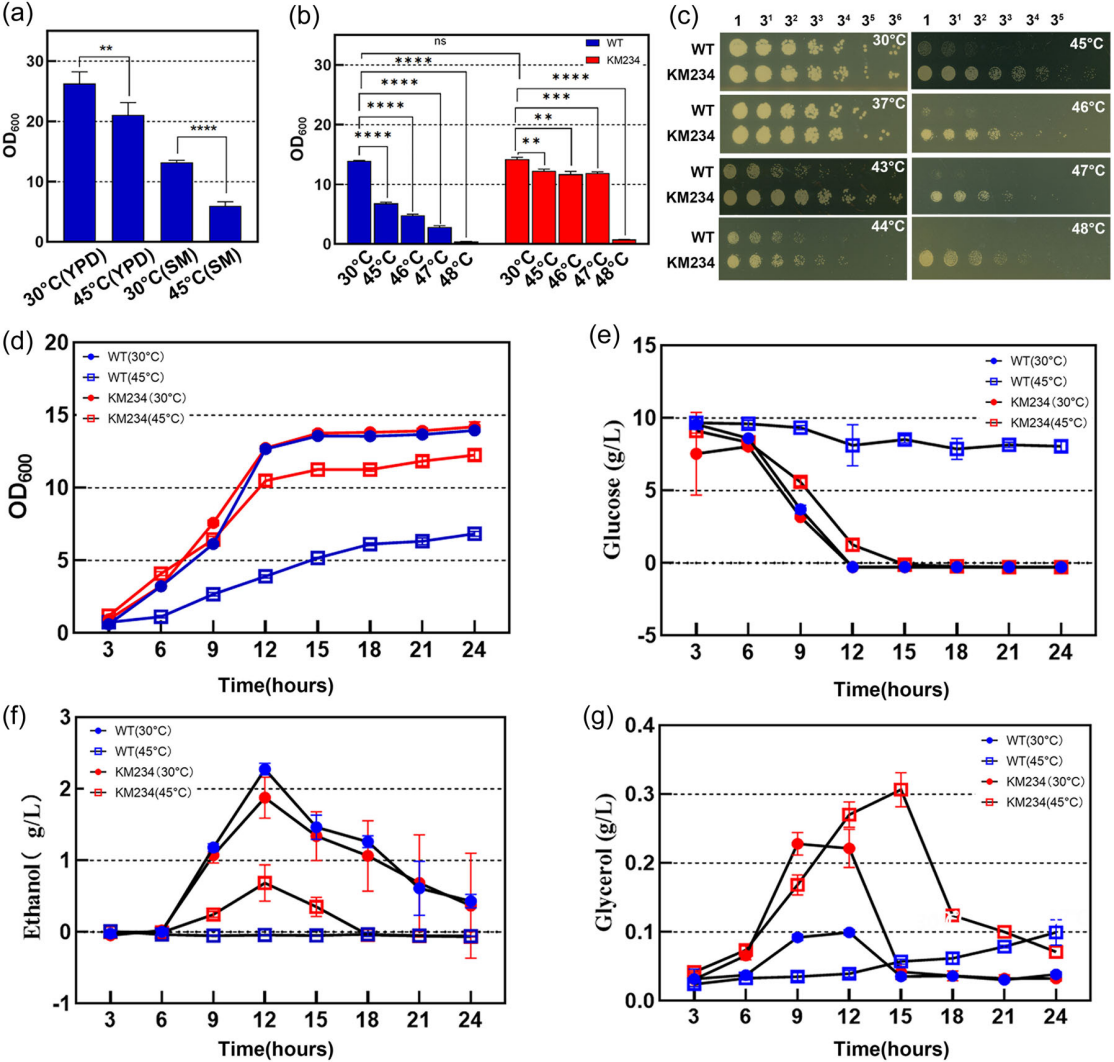
Growth phenotypes of the wild‐type (WT) *Kluyveromyces marxianus* and the evolved strain KM234. (a) The cell densities optical densities at 600 nm (OD_600_) of the WT strain cultured at 30°C and 45°C for 24 h in peptone dextrose medium (YPD) and synthetic medium (SM), respectively. (b) Growth of the WT and KM234 strains at different temperatures in SM. Both strains were grown at 30°C, 45°C, 46°C, 47°C, and 48°C in SM flasks shaking at 220 rpm for 24 h. (c) The spot assay of the WT and KM234 strains on YPD plates at different temperatures. Cell cultures grown in YPD liquid medium overnight were adjusted to an OD_600_ of 1.0, and then serially diluted with sterilized water. From each dilution, 3 μL aliquots were spotted on YPD plates. The spotted plates were incubated at the indicated temperatures for 16–24 h before imaging. Determinations of the time courses of cell densities (d) and glucose consumption rates (e), and concentrations of ethanol (f) and glycerol (g) for the WT and KM234 strains grown at 30°C and 45°C in SM. Values are means with standard deviations (*n* = 3) and the *p*‐values were calculated by the *t*‐test. *p < .05; **p < .01; ***p < .001; ****p < .0001.

A distinct difference between the complete medium and minimal medium for yeast growth is the form of nitrogen source. The effect of media on the wild‐type (WT) *K. marxianus* growth at high temperatures was first evaluated using the common laboratory medium YPD and SM defined for the industrial fermentation of *K. marxianus*. As shown in Figure 1a, compared to 30°C, the cell density OD_600_ of the WT strain incubated at 45°C for 24 h in YPD decreased by 19.89%, while it decreased by 54.8% in SM medium under the same culture conditions. These results demonstrated that the thermotolerance of *K. marxianus* significantly decreased in the SM medium. This phenotype seems to be a general physiological feature for *K. marxianus* after testing different *K. marxianus* strains including ATCC26548 and NBRC1777.

Accordingly, ALE of the WT strain was performed at 45°C in SM. Among 135 clones derived from the 100‐day ALE, a mutant strain, named KM234, exhibited high growth ability than the WT strain at high temperatures. This was further verified by the growth tests at gradient temperatures. As shown in Figure 1b, the growth of the KM234 strain was not significantly different from the WT strain when cultured at 30°C in SM, but at 45°C, its cell density OD_600_ was about twice that of the WT strain. In particular, at 47°C, the cell density OD_600_ of the KM234 strain was 4.2 times that of the WT strain. The spot assay likewise showed a significant difference in growth between the WT strain and the KM234 strain in the YPD medium at high temperatures. As shown in Figure 1c, the KM234 strain still grew well at 48°C, whereas the WT strain could hardly grow at temperatures over 44°C. These results demonstrate that the KM234 strain has significantly improved its high‐temperature tolerance without obvious loss of growth ability at a moderate temperature.

Metabolites accumulated intracellularly and extracellularly may have a great effect on yeast thermotolerance. In yeast, ethanol and glycerol are two major metabolites of the overflow metabolism that occurred when the glucose uptake rate exceeds a threshold rate (Vemuri et al., 2007). Thus, the glucose, ethanol, and glycerol concentrations in media were detected at an interval of 3 h during culturing of both strains in SM at 30°C and 45°C. As shown in Figure 1d–f, there were no significant differences between the two strains in glucose consumption rates, as well as ethanol production, when grown at 30°C in SM. Growing at 45°C, the KM234 strain had a higher glucose consumption rate and produced lower than 0.8 g/L ethanol during the period of 6–8 h. In the case of the WT strain, however, >2 g/L glucose had been consumed, but no ethanol was produced, which is probably due to its poor growth at this temperature. Unlike glucose and ethanol, which are generally considered pro‐aging carbon sources, glycerol contributes to protecting yeast against environmental stress and promoting life span extension (Wei et al., 2009). Compared to the WT strain, the KM234 strain produced a higher amount of glycerol during its rapid growth period at either 30°C or 45°C. It seems that the increased glycerol production by the KM234 stain contributes to protecting it against high‐temperature stress (Fu et al., 2019). This assumption is further supported by the subsequent RNA‐Seq analysis.

### 3.2 Global changes in the genomes and transcriptomes between the WT and KM234 strains

To check the genome changes that may be responsible for the phenotypes of high‐temperature tolerance, the genome of the KM234 strain was resequenced. There were 16 mutations identified in the KM234 genome, including 11 SNPs and 5 INDELs (Table 1). Among them, only six SNPs are located in the gene coding regions of *EXG1*, *KIN1*, *FIM1_2659*, and *PDR5*. *EXG1* is an exo‐1, 3‐β‐glucanase gene that implicates in the biosynthesis of cell wall β‐glucan. *KIN1* encodes an S/T protein kinase, belonging to a subfamily of the adenosine monophosphate (AMP)‐activated kinase‐related kinases (AMPKRs), that regulates polarized exocytosis and the Ire1p‐mediated unfolded protein response. And, *FIM1_2659* is a hypothetical zinc finger protein. In *S. cerevisiae*, *PDR5* encodes a major multidrug resistance efflux pump. The five INDELs exist in the *GIS3*, *IOC2*, *HMS1*, *LMO1*, and *PDE2* genes, which code for two hypothetical proteins, two transcription factors, and a high‐affinity cyclic AMP phosphodiesterase, respectively.

**TABLE 1:** SNPs and INDELs in the genome of the KM234 strain.

Genes	Mutation sites	Gene locations	Mutant types		Gene functions
**SNPs**					
*EXG1*	1261479	CP015054.1	GGG/Gly	UGG/Trp	Glucan 1,3‐beta‐glucosidase Exg1
*KIN1*	371998	CP015057.1	GAG/Glu	GUG/Val	Serine/threonine protein kinase Kin1
*FIM1_2659*	478073	CP015057.1	GGG/Gly	UGG/Trp	Zinc finger protein YPR013C
	583922		GCT/Ala	UGG/Trp	
*PDR5*	583934	CP015057.1	GGA/Gly	GGG/Gly	A major multidrug resistance efflux pump
	583958		CCT/Pro	CCC/Pro	
*LAC12*	38753	CP015056.1	cccgccaaaa	cccgccaaaaa	Lactose permease/high‐affinity glucose transporter
*GZF3*	556453	CP015057.1	taaa	Aaaa	Gzf3p, GATA‐binding protein
*MDM31*	1147628	CP015057.1	ttaaa	ttaat	Mitochondrial distribution and morphology protein 31
*DBP2*	705872	CP015059.1	attttt	tttttt	DEAD‐box ATP‐dependent RNA helicase Dbp2
*RPC19*	705872	CP015059.1	attttt	tttttt	DNA‐directed RNA polymerase core subunit Rpc19
*AMD2*	1679057	CP015055.1	gag	ggg	putative amidase
**INDELs**					
*PDE2*	1272468	CP015056.1	Insert	T	3',5'‐cyclic‐nucleotide phosphodiesterase
*HMS1*	986441	CP015059.1	Deletion	G	protein similar to myc‐family transcription factors
*LMO1*	505189	CP015056.1	Insert	AAAAAA	Lmo1p, signaling protein involved in mitophagy
*GIS3*	364985	CP015057.1	Deletion	A	hypothetical proteins
*IOC2*	364985	CP015057.1	Deletion	A	hypothetical protein

**FIGURE 2:**
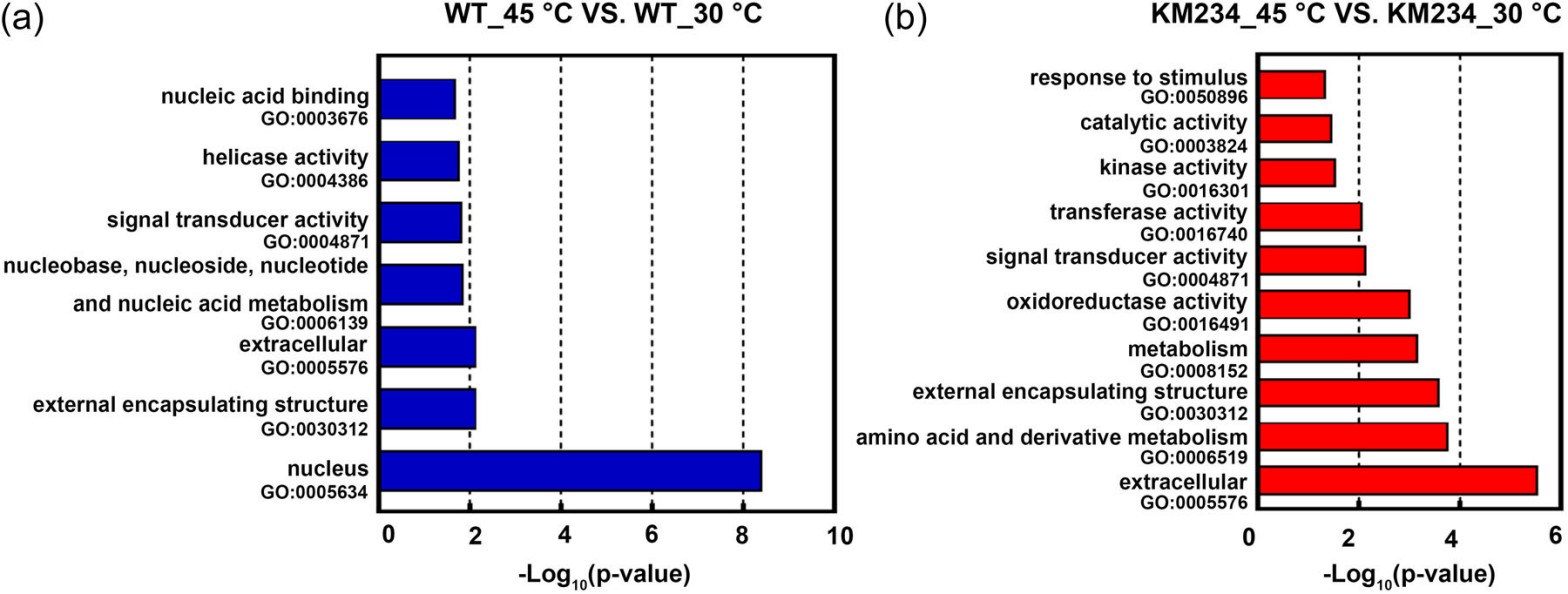
Gene Ontology (GO) clusters of the differentially expressed genes in the WT_45°C versus WT_30°C (a) and KM234_45°C versus KM234_30°C (b) under high‐temperature stress. For RNA‐seq, the wild‐type (WT) and the KM234 strains were grown in synthetic medium (SM) at 30°C and 45°C, respectively, and yeast cells were harvested after 8 h. Genes with adjusted *p* values <0.05 or the log2 (fold change) values >1 were assigned as differentially expressed.

RNA‐seq was also conducted to analyze the transcriptomes of the WT and KM234 strains. The raw data have been submitted to the NCBI BioProject under the accession number PRJNA782397. At 30°C, there were 86 upregulated genes and 546 downregulated genes in the KM234 strain compared to the WT strain. In WT_45°C, 792 genes were upregulated, and 130 genes were downregulated compared with WT_30°C. At KM234_45°C, 720 genes were upregulated, and 176 genes were downregulated compared with KM234_30°C. There were 325 upregulated genes and 1184 downregulated genes in KM234_45°C vs WT_45°C. Gene Ontology (GO) analyses showed that the differential expressed genes were different in the WT and KM234 strains in responding to the high‐temperature stress. In the clustering of WT_45°C vs WT_30°C, GO terms of nucleus‐related genes were enriched significantly (Figure 2a) into nucleus‐related metabolism, nucleobase, nucleoside, nucleotide, and nucleic acid metabolism, nucleic acid binding, helicase activity, in addition to external encapsulating structure, extracellular and signal transducer activity. In the KM234 strain, besides the genes in the GO terms of signal transducer activity, extra encapsulating structure, and extracellular, genes for amino acid and derivative metabolism, stimulus, and oxidoreductase activity were also differentially expressed (Figure 2b).

### 3.3 Expressions of the NH_4_^+^ transporter genes were downregulation and expressions of amino acid carbon skeleton synthesis pathway genes were upregulated in the KM234 at high temperatures

**FIGURE 3:**
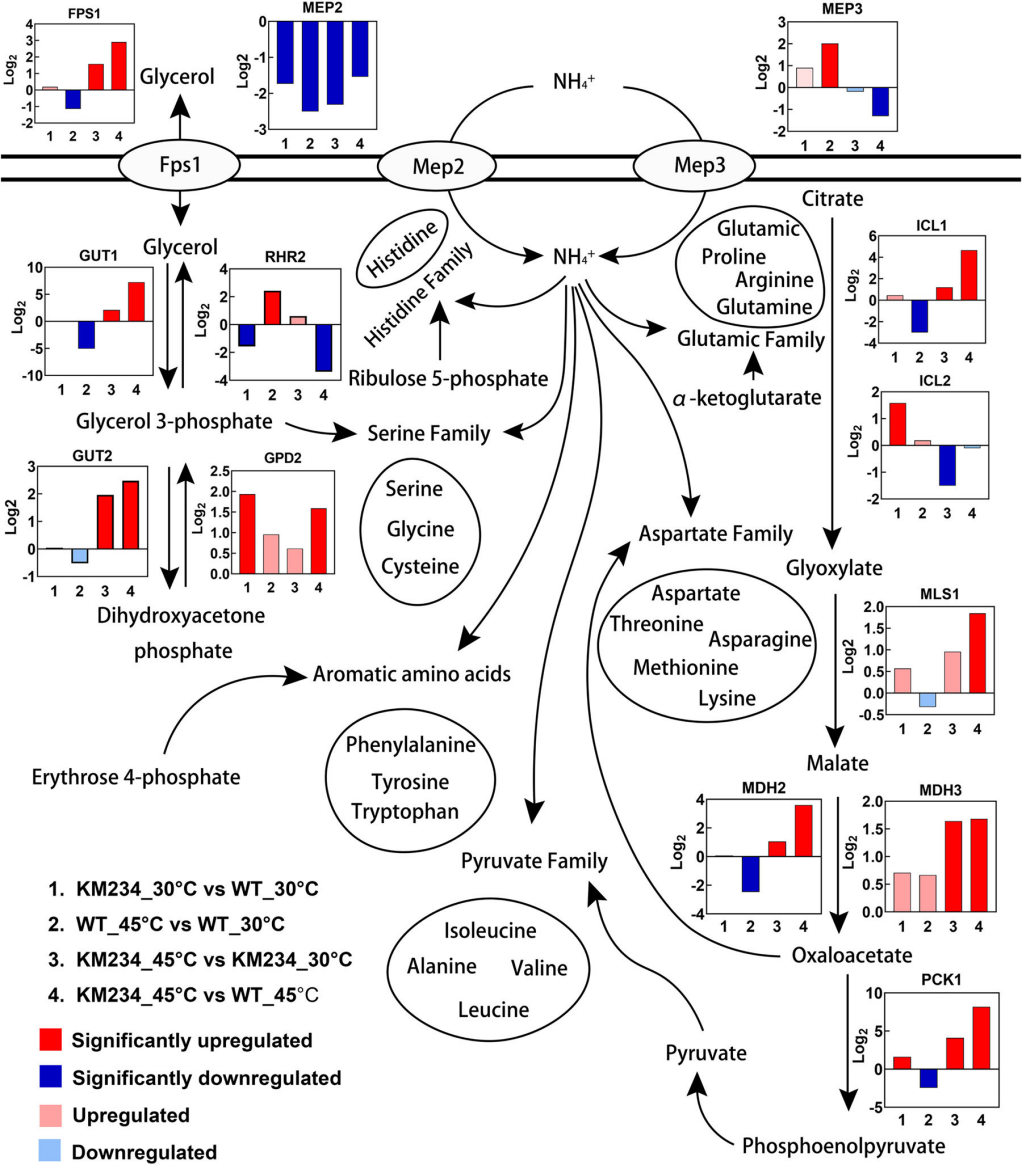
Transcript profiles of the genes involved in ammonium uptake and amino acid carbon skeleton synthesis carbon metabolism pathways in the wild type (WT) and KM234 strains under different temperatures*.* The fold changes of the gene transcriptions between different temperatures or different strains were illustrated as log2 (fold change) values. The histogram represented the change in the specific gene expressions.

At 45°C, the top 10 significantly upregulated genes in the KM234 strain were mostly enriched in the gluconeogenesis, glyoxalate cycle, and glycerol metabolism pathways (Table 2), which also are upstream pathways for the synthesis of amino acid carbon skeletons (Figure 3). The *GUT1*, *GUT2*, *FPS1*, and *GPD2* genes involved in glycerol uptake and metabolism were upregulated in the KM234 strain when grown at 45°C. By contrast, the *RHR2* gene, which encodes a glycerol‐3‐phosphatase responsible for the terminal step in glycerol biosynthesis from glycerol‐3‐phosphate (Fan et al., 2005), was downregulated. This was consistent with the above result that the KM234 strain accumulated glycerol during log‐phase growth, and metabolized it as the carbon resource after glucose was depleted (Figure 1e). It is worth noting that glycerol‐3‐phosphate is an important carbon skeleton precursor for the synthesis of serine family amino acids and sterols. Consequently, downregulation of the *RHR2* gene is conducive to the synthesis of downstream amino acids.

**TABLE 2:** Top 10 upregulated genes in the cluster of KM234_45°C vs WT_45°C.

Genes	Predicted protein functions	Log2 (fold_changes) normalized	Related pathways
*PCK1*	Phosphoenolpyruvate carboxykinase	8.1765	Gluconeogenesis
*GUT1*	Glycerol kinase	7.2584	Glycerol metabolism
*FBP1*	Fructose‐1‐6‐bisphosphatase	5.9110	Gluconeogenesis
*SIP4*	Protein SIP4	5.6456	Gluconeogenesis promoting factor
*ALD6*	Magnesium‐activated aldehyde dehydrogenase	4.9988	Ethanol metabolism
*JEN1*	Carboxylic acid transporter protein‐like protein	4.8326	Carbon source transport
*ICL1*	Isocitrate lyase	4.6732	Glyoxylate pathway
*‐*	Hypothetical protein	3.8871	‐
*ALD5*	Aldehyde dehydrogenase 5	3.8559	Ethanol metabolism
*MDH2*	Malate dehydrogenase 2	3.6029	Glyoxylate Pathway/TCA cycle

**TABLE 3:** Primer sequences and gRNA used in this study.

Primers	Sequences
UhfKIN1F	CCCTGTCTAACGCCATTATGTACG
UhfKIN1R	CACTGAATTGGTTCAACAAATCTTATTCTAGCTAATCTAATCCTCAACCACCT
DhfKIN1F	AGGTGGTTGAGGATTAGATTAGCTAGAATAAGATTTGTTGAACCAATTCAGTG
DhfKIN1R	GTTGATTCGATACCCTAGACCTGAT
KIN1DvF	TAACAGTACATACACAATCGGTGCC
KIN1DvR	CGTTTATGCAGAGAAGCAAAGAAG
gRNA	ATCAGCTAGAATGCCATCAGCTC

In addition, genes involved in the glyoxylate pathway were upregulated in the KM234 strain, especially the isocitrate lyase gene *ICL1* and malate synthase gene *MLS1*. Oxaloacetate is a precursor for the synthesis of aspartic acid‐like amino acids, and the *PCK1* gene, encoding the phosphoenolpyruvate carboxykinase1 that catalyzes the synthesis of phosphoenolpyruvate from oxaloacetate, was also upregulated significantly. The phosphoenolpyruvate product can be directly used for the synthesis of phenylalanine, tryptophan, and tyrosine together with 4‐phospho‐erythrose (Lea et al., 2001), or serves as a precursor for the syntheses of pyruvate and downstream alanine, valine, leucine, and isoleucine.

In *S. cerevisiae*, Mep1 and Mep3 are high flux, low‐affinity NH_4_^+^ transporters, while Mep2 is a low flux, high‐affinity transporter (Marini et al., 1997). However, there are only two transporters, Mep2 and Mep3, in *K. marxianus*. From RNA‐seq, the *MEP2* gene was significantly downregulated in both the WT and KM234 strains at high temperatures. More importantly, its expression in the KM234 train was significantly lower than in the WT strain at 30°C. By contrast, expression of the *MEP3* gene was only significantly upregulated in the WT strain when grown at 45°C. This finding indicates that downregulating the expression of NH_4_^+^ transporter in the KM234 strain is beneficial to the growth of *K. marxianus* at high temperatures. We, therefore, speculated that the KM234 strain had evolved an ability to repress NH_4_^+^ uptake by controlling the expression of transporter genes against high‐temperature stress.

### 3.4 The dual effects of ammonium affecting the yeast growth at high temperatures

**FIGURE 4:**
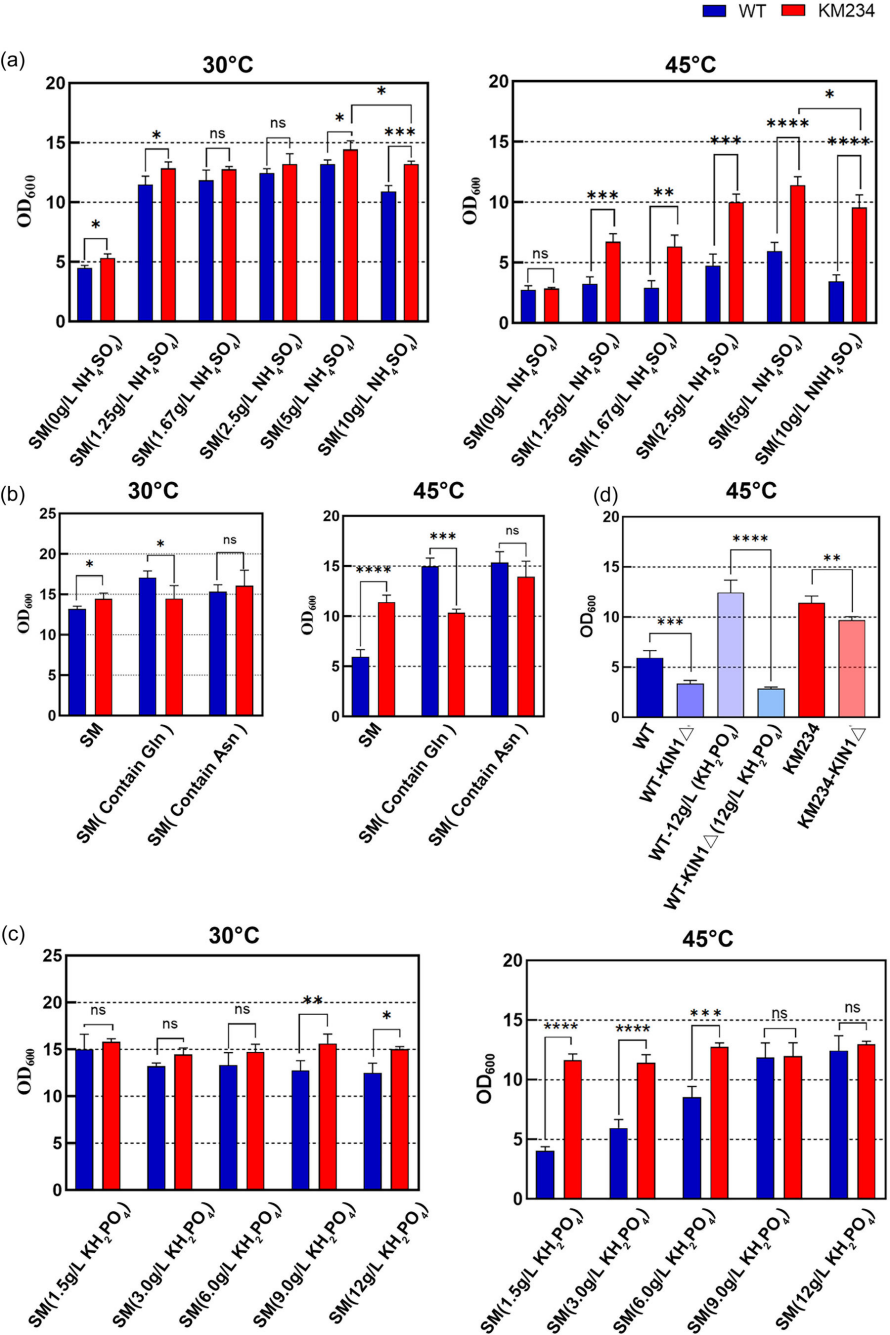
The specific toxicity of ammonium as a nitrogen source at high temperatures. (a) Influence of ammonium in synthetic medium (SM) on the growth ability of *Kluyveromyces marxianus* at 30°C and 45°C. The cell densities of the wild type (WT) and the KM234 strains were recorded for 24 h in SM containing 0–10 g/L (NH_4_)_2_SO_4_. (b) The cell densities of the WT and the KM234 strains grown in SM at 30°C and 45°C for 24 h, in which (NH_4_)_2_SO_4_ was replaced by the same concentration (g/L) of glutamine or asparagine. (c) Effects of K^+^ concentrations on the growth abilities of the WT and KM234 at different temperatures. Cell densities of the WT and the KM234 strains grown at 30°C and 45°C were recorded for 24 h in SM containing 1.5–12 g/L KH_2_PO_4_. However, for the WT‐*KIN1*Δ and KM234 Δ*KIN1* strains, only 12 g/L of KH_2_PO_4_ was assayed. *p < .05; **p < .01; ***p < .001; ****p < .0001; ns, no significant difference. (d) Effects of the *KIN1* deletion on the growth of the WT, the KM234, the WT‐*KIN1*Δ, and KM234‐*KIN1*Δ strains at 45°C.

To further verify the connection between NH_4_^+^ and high‐temperature growth, we tested the effect of the (NH_4_)_2_SO_4_ contents in the medium on yeast growth. As shown in Figure 4a, increasing the (NH_4_)_2_SO_4_ concentration in SM enhanced the growth of both the WT and KM234 strains if it was not higher than 5 g/L. However, when the (NH_4_)_2_SO_4_ content was increased to 10 g/L, twice the normal concentration in SM, the cell densities of the WT strain decreased by 17.3% at 30°C and 42.1% at 45°C, respectively. By contrast, the KM234 strain, decreased only by 8.6% at 30°C and 16.4% at 45°C, respectively. These results indicate that excess NH_4_^+^ inhibits yeast growth even at moderate temperatures. The negative effect of ammonium on the growth of the WT strain became more severe at high temperatures, whereas the KM234 strain has significantly increased its toxic tolerance to NH_4_^+^ after evolution. This finding is consistent with the RNA‐seq results that both the NH_4_^+^ transporter genes *MEP2* and *MEP3* are significantly downregulated in the KM234 strain when grown at high temperatures.

As ammonium sulfate is the sole source of nitrogen in SM, insufficient NH_4_^+^ may cause poor growth of yeast, which interferes with its behavior in affecting the thermotolerance of yeast. Nevertheless, the NH_4_^+^ toxicity can be alleviated by replacing ammonium sulfate with organic nitrogen asparagine (Hess et al., 2006). Given this, we replaced ammonium sulfate in SM with 5 g/L glutamine (Gln) or asparagine (Asn). As a result, substitution with Gln or Asn in SM substantially rescued the growth defect of the WT strain at 45°C but had no apparent effect on the growth of the KM234 strain at high temperatures (Figure 4b). This result was in agreement with our previous speculation that the KM234 strain had evolved an adaptive mechanism to avoid uptake of excess NH_4_^+^ at high temperatures.

In yeast, NH_4_^+^ uptake competes with K^+^ transportation, and increasing K^+^ concentration can inhibit NH_4_^+^ uptake and mitigate the toxicity of high NH_4_^+^ concentration (Hess et al., 2006). This competition may help to explain how the KM234 strain enhanced its thermotolerance. Therefore, we varied the K^+^ content in SM and tested the growth phenotypes of the WT and KM234 strains. At 30°C, increasing the KH_2_PO_4_ concentrations from 1.5 to 12 g/L slightly decreased the growth of the WT strain, but has no apparent effect on that of the KM234 strain (Figure 4c). On the contrary, the OD_600_ values of the WT cells increased from 4.02 to 12.45 as the KH_2_PO_4_ contents in SM increased from 1.5 to 12 g/L when grown at 45°C. Notably, in SM containing 9 g/L KH_2_PO_4_, the cell density of the WT strain grown at 45°C was comparable to that of the KM234 strain. Similar to that at 30°C, changes in the KH_2_PO_4_ content in SM had no significant effect on the growth of the KM234 strains at high temperatures.

Since an SNP occurred in the *KIN1* gene of the KM234 strain, Kin1 can phosphorylate Pal2 to modulate the splicing of HAC1 mRNA, which is a key transcriptional factor initiating the transcriptions of unfolded protein response (UPR) target genes to cope with the stress caused by the accumulation of misfolded proteins in the endoplasmic reticulum (Travers et al., 2000), we deleted the *KIN1* gene in both the WT and KM234 strains. Deletion of the *KIN1* gene significantly reduced the growth of both strains at high temperatures (Figure 4d) by 34.0% and 15.6%, respectively. The growth of the WT‐*KIN1*Δ strain was also significantly decreased by 72.8% at high temperatures in SM containing 12 g/L of KH_2_PO_4_. This result implies that the alleviation of NH_4_^+^ toxicity by K^+^ is greatly dependent on the presence of *KIN1*.

### 3.5 Ammonium affects the high‐temperature growth of yeast by producing ROS

**FIGURE 5:**
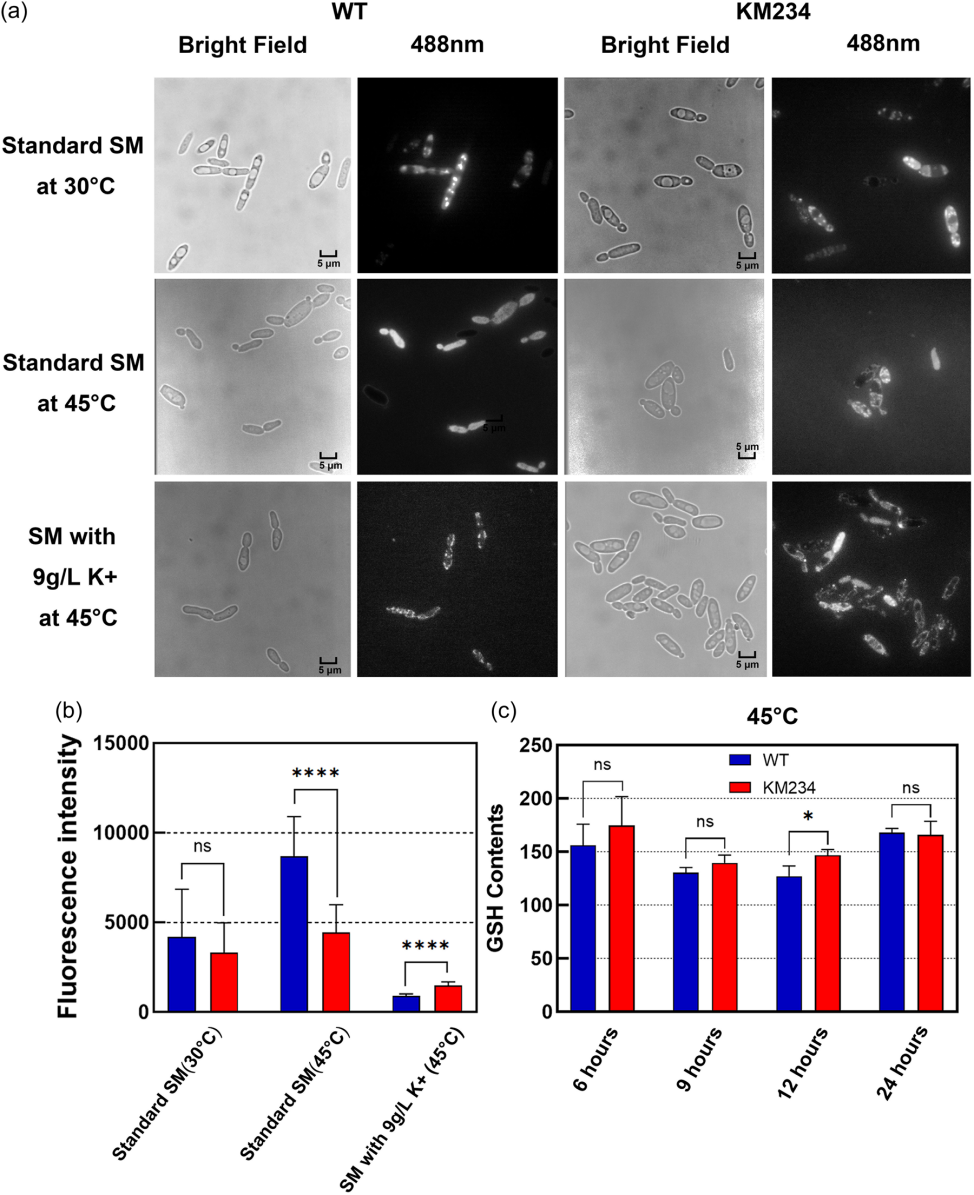
The reactive oxygen species (ROS) levels of the wild type (WT) and KM234 strains in the logarithmic phase. (a) ROS fluorescences in 8 h old *Kluyveromyces marxianus* cells. Bars, 5 μm. (b) The ROS fluorescence intensities of 8‐h‐old yeast cells grown at 30°C and 45°C in synthetic medium (SM) or with 9 g/L KH_2_PO_4_. (c) The glutathione contents in both strains grown at 45°C for different time periods. Values were means ± SE from four independent replicates. *p < .05; ****p < .0001; ns, no significant difference.

Heat usually stimulates yeast to produce more harmful reactive oxygen species (ROS) that can affect its growth at high temperatures (Mejía‐Barajas et al., 2017). Excess NH_4_^+^ also induces the production of ROS in yeast (Yang et al., 2020). To investigate whether the loss of growth ability at high temperatures in SM was ascribed to the high level of ROS, ROS in both strains under different temperatures were determined by a fluorescent probe (Yang et al., 2020). As shown in Figure 5a, ROS fluorescences in the WT and KM234 cells, cultured in standard SM at 30°C, showed dot‐like distributions, which suggests that they produce low ROS levels when grown at moderate temperature. At 45°C, ROS fluorescence in the KM234 cells was still distributed punctiform, but it filled the whole cells of the WT strain. Moreover, if the WT strain grew at 45°C in SM containing 9 g/L KH_2_PO_4_, ROS fluorescence in cells was the same as when it grew at 30°C. These findings indicate that the KM234 strain might accumulate a lower level of ROS than the WT strain when grown at a high temperature. To validate this, we quantified the ROS contents in cells for both strains. As shown in Figure 5b, there was no significant difference in the ROS contents between the two strains grown at 30°C. Although the ROS contents were substantially increased in both strains at 45°C, the WT strain accumulated a significantly higher ROS content than did the KM234 strain when grown in SM both at 30°C and 45°C. If grown in SM contained high K^+^ content the result was the opposite. In addition, ROS contents in both strains were significantly decreased when grown at 45°C in the presence of 9 g/L KH_2_PO_4_. These results suggest that excess NH_4_^+^ induces the production of ROS in SM. In addition to ROS from high‐temperature stress, yeast needs to cope with more ROS than organic nitrogen source alone, which may exceed the handling capacity of yeast and leads to the loss of growth ability at high temperatures.

GSH is one of the major free radical scavengers that maintains the intracellular redox balance. As ROS burst is usually accompanied by an increase in GSH production, we measured the intracellular GSH contents in both strains grown at 45°C. As shown in Figure 5c, except that the GSH content in the KM234 strain was significantly higher than in the WT strain at 12 h, there was no significant difference in the intracellular GSH between the two strains. These results suggest that the GSH synthesis pathway in the KM234 strain has not been changed after ALE.

## 4 DISCUSSION

Nitrogen provides an essential element for all forms of life. It is also a structural component for the synthesis of basic macromolecules such as nucleic acids, proteins, and other types of molecules, for example, alkaloids and thioglucosides (Landi et al., 2019). The main organic nitrogen sources in nature are urea and allantoin, amino acids, short peptides, and proteins (Lea et al., 2001). Inorganic nitrogen sources include inorganic compounds such as ammonium (NH_4_^+^), nitrates (NO_3_^−^), nitrites (NO_2_^−^), and nitrogen gas. Nitrogen metabolism in a cell is precisely regulated and insufficient supply will lead to poor growth (Zhang et al., 2018), but excess ammonium can bring toxicity (Vidotto et al., 1993). For example, in plants, nitrogen fertilizers are rapidly converted to NH_4_^+^ by urease, which provides nitrogen nutrients for their growth. However, excess NH_4_^+^ leads to the accumulation of large amounts of ROS that can affect plant growth (Yang et al., 2020). Similarly, high levels of NH_4_^+^ in human blood cause hyperammonemia (Santos et al., 2012). So, NH_4_^+^ is a preferred source of inorganic nitrogen for yeast, and also acts as a “negative factor” implicated in the ROS production in the senescent yeast cells, as well as the regulation of the chronological lifespan (CLS) (Santos et al., 2015). Although excess NH_4_^+^ has been proved to be cytotoxic, little is known about the effect of NH_4_^+^ on the thermotolerance and growth ability of yeast at high temperatures. This study demonstrates for the first time the toxicity of ammonium to *K. marxianus* at high temperatures when using inorganic nitrogen as the sole nitrogen source. Uptake of excess NH4^+^ promotes the production of ROS and adversely impacts the growth ability of *K. marxianus*.

How does NH_4_^+^ affect the thermotolerance of yeast? Probably, this is mainly due to the ROS production promoted by both heat and NH_4_^+^ when incubated at high temperatures in SM. If the total amount of intracellular ROS exceeds the antioxidant defense system, the yeast cell is in a status of oxidative stress that may drastically reduce its growth ability. At high temperatures, ROS is rapidly generated by the protons leaked from the electron transport chain (ETC) and caused damage to proteins, lipids, and DNA (Zorov et al., 2014). To respond the oxidative stress, *K. marxianus* need to increase the NAPDH output via upregulating the pentose phosphate pathway (PPP) and reduce the NADH output and consumption by downregulating the TCA cycle and ETC pathway when grown in YPD (Lertwattanasakul et al., 2015). In this study, however, the oxidative phosphorylation pathway that is closely related to ATP production, as well as the PPP pathway, were not significantly changed when grown in SM at high temperatures. Excessive intake of NH_4_^+^ triggers ROS production (Yang et al., 2020), and removal of NH_4_^+^ or replacement with amide amino acids can extend the CLS of yeast (Santos et al., 2015). The most obvious difference between YPD and SM is the form of nitrogen resources, and two NH_4_^+^ transporter genes *MEP2* and *MEP3* were both downregulated in the evolved strain (KM234_45°C vs WT_45 °C), we then speculate that NH_4_^+^ is a critical factor in the thermotolerance of *K. marxianus*. In addition, under K^+^‐limited conditions, high NH_4_^+^ concentration impairs the growth of brewer's yeast, and if the NH_4_^+^ transporter is constitutively expressed, NH_4_^+^ can cause damage even in the presence of a high concentration of K^+^ (Hess et al., 2006; Shi et al., 2020). Therefore, to confirm our speculation, nitrogen resource substitution and competition assays with K^+^ were conducted. Consistent with the previous study, replacing the nitrogen source with glutamine or asparagine restored the growth ability of the WT strain at high temperatures, and a similar result was obtained by increasing the K^+^ concentration in SM. However, nitrogen resource substitution and high concentration of K^+^ had not brought apparent effects on the growth of the evolved strain. These results suggest that the evolved strain may decrease the NH_4_^+^ uptake, avoiding the toxicity of excess NH_4_^+^ at high temperatures. If necessary, NH_4_^+^ toxicity to WT *K. marxianus* can be eliminated by a high ratio of K^+^/NH_4_^+^, and this is a practical way for industrial fermentations under high‐temperature conditions.

In plants, excess NH_4_^+^ causes a burst of ROS and leads to redistributing the carbon metabolic flux, for example, remarkably upregulates glycolytic and glycogenolytic pathways, and increases carbon skeleton synthesis as well as GSH synthesis (Yang et al., 2020). However, we found the GSH amount was not changed, but transcriptome analysis revealed that the carbon metabolism was redirected to respond to the high‐temperature stress in *K. marxianus*, with more carbon directed to synthesize the amino acid carbon skeleton. The glycerol metabolic pathway, glyoxylate pathway, and gluconeogenesis pathway were synchronously upregulated in the evolved strain grown at high temperatures. Unlike yeast*,* the plant generally upregulates the glyoxylate pathway during seed germination to supply lipogenic carbon sources for growth, and the transcriptions of gluconeogenesis genes are concomitantly upregulated (Graham, 2008). In the KM234 strain, glyoxylate and gluconeogenesis pathways were simultaneously upregulated during the logarithmic phase (8 h). However, as shown in Figure 1e, glucose was sufficient during this phase. Thus we supposed that upregulating gluconeogenesis in *K*. *marxianus* at high temperatures tended to produce more intermediates for other metabolic pathways, not to replenish carbon sources. This is because the glyoxylate pathway is a faster pathway for oxaloacetate production than the TCA cycle, which is conducive to synthesizing amino acids of the aspartate family, such as aspartic acid, asparagine, threonine, methionine, and lysine (Figure 3). To assimilate inorganic nitrogen, yeast also needs to constantly synthesize carbon skeletons that are mainly from the intermediates of sugar metabolism (Maslanka & Zadrag‐Tecza, 2020). Therefore, it is reasonable that *PCK1*, a key enzyme for sugar gluconeogenesis, is the most significantly upregulated gene in the KM234 strain at high temperatures.

Yeast Kin1 is a serine/threonine kinase located on the cell plasma membrane surface (Lamb et al., 1991; Tibbetts et al., 1994). Current studies have not yet mentioned that it participates in NH_4_^+^ uptake, K^+^ transport, and high‐temperature tolerance. In the KM234 strain, the *KIN1* gene contains an SNP that an acidic amino acid glutamate is changed to nonpolar valine. Knockout of the *KIN1* gene reduced the growth ability of *K. marxianus* at high temperatures. More importantly, the loss of the *KIN1* gene has a higher impact on the growth of the WT strain than the KM234 strain, and the high ratio of K^+^/NH_4_^+^ cannot rescue the growth defect of the WT‐*KIN1*Δ strain. As described above, to cope with the high‐temperature stress, the KM234 strain decreased its NH_4_^+^ uptake by downregulation of NH_4_^+^ transport genes and increasing the synthesis of the amino acid carbon backbone. In WT *K. marxianus*, ammonium toxicity can be mitigated by the replacement of ammonium sulfate with amide amino acids or increasing the K^+^ content in SM, while the latter approach relies on the gene *KIN1*, which may act similarly to *CIPK23* in *Arabidopsis* (Shi et al., 2020). However, the underlying mechanism for the regulation of the NH_4_^+^ transport genes including *MEP2* and *MEP3* by *KIN1* needs to be investigated in a further study.

## 5 CONCLUSIONS

Ammonium regulates the growth of *K. marxianus* in the SM, and uptake of excess NH4^+^ promotes the production of ROS and decreases the growth ability of *K. marxianus* at high temperatures. A high ratio of K^+^/NH_4_^+^ rescues the growth defect of the WT strain at high temperatures, and the serine/threonine kinase *Kin1* may participate in this regulation. An increase in K^+^ content can alleviate the ammonium toxicity and significantly increase the high‐temperature growth ability of *K. marxianus* in the SM. Our result provides a practical way for *K. marxianus* in industrial fermentations at high temperatures.

## AUTHOR CONTRIBUTIONS

**Yi Ai**: Data curation (equal); formal analysis (equal); investigation (equal); methodology (equal); validation (equal); visualization (equal); writing—original draft (equal). **Tongyu Luo**: Data curation (equal); formal analysis (equal); investigation (equal); methodology (equal); resources (equal). **Yao Yu**: Formal analysis (equal); methodology (equal); writing—review and editing (equal). **Jungang Zhou**: Project administration (equal); validation (equal); writing—original draft (equal); writing—review and editing (equal). **Hong Lu**: Conceptualization (equal); project administration (equal); resources (equal); funding acquisition (equal); supervision (equal); writing—original draft (equal); writing—review and editing (equal).

## CONFLICT OF INTEREST

None declared.

## ETHICS STATEMENT

None required.

## Data Availability

All data are provided in full in this paper apart from the raw data of transcriptome sequencing, which are available at the NCBI database under the BioProject PRJNA782397: https://www.ncbi.nlm.nih.gov/bioproject/PRJNA782397. The details for the differentially expressed genes enriched in GO clusters are available in the Zenodo repository at https://doi.org/10.5281/zenodo.6496417.
